# Radiographic Clarity of the External Auditory Meatus as a Cranial Reference Point in Spinal Deformity Patients: A Pilot Interobserver Study

**DOI:** 10.3390/jcm15082971

**Published:** 2026-04-14

**Authors:** Dongkyu Kim, Dong Kyu Chin, Sejun Park, Jaemin Kim, Insu Lee, Jun-Woo Ha, Hyun Jun Jang, Kyung Hyun Kim, Jeong Yoon Park, Sung Uk Kuh, Keun Su Kim, Pyung Goo Cho, Bong Ju Moon

**Affiliations:** 1 Department of Neurosurgery, The Spine and Spinal Cord Institute, Gangnam Severance Hospital, Yonsei University College of Medicine, Seoul 06273, Republic of Korea; kyu0214@yuhs.ac (D.K.); dkchin@yuhs.ac (D.K.C.); dannypsj@yuhs.ac (S.P.); jmns333@yuhs.ac (J.K.); derrra@yuhs.ac (I.L.); jwh20@yuhs.ac (J.-W.H.); jangjh0@yuhs.ac (H.J.J.); nskhk@yuhs.ac (K.H.K.); spinepjy@yuhs.ac (J.Y.P.); kuhsu@yuhs.ac (S.U.K.); spinekks@yuhs.ac (K.S.K.); 2 Department of Neurosurgery, Ajou University Medical Center, Suwon-si 16499, Republic of Korea

**Keywords:** cranial center, external auditory meatus, sella turcica, spinal deformity

## Abstract

**Background/Objectives**: The external auditory meatus (EAM) is widely used as a surrogate cranial reference point, based on its proximity to the midpoint of the nasion–inion line (MNI). However, its radiographic clarity has not been thoroughly validated. This study aimed to evaluate the radiographic clarity and interobserver reproducibility of the EAM compared with the sella turcica as a control landmark. **Methods**: A retrospective review was performed on patients who underwent surgical correction for sagittal spinal deformity between 2021 and 2024. Preoperative standing whole-spine radiographs were analyzed. Horizontal and vertical distances from the EAM and the posterior border of the sella turcica to the MNI were measured. Radiographic clarity was categorized into three groups. Five independent neurosurgeons conducted all measurements, and interobserver reliability was assessed using the intraclass correlation coefficient with a two-way random-effects model [ICC(2,1)]. **Results**: The EAM was horizontally closer to the MNI (1.1 mm vs. 13.8 mm) but exhibited poorer radiographic clarity, with only 14.1% classified as single point and clear compared with 84.5% for the sella turcica. Interobserver reproducibility was lower for the EAM (ICC: 0.84 horizontal, 0.89 vertical) than for the sella turcica (0.97, 0.95). Horizontal deviation among observers was significantly greater for the EAM (major deviation 6.3 mm vs. 2.2 mm, *p* < 0.001), whereas vertical deviation did not differ significantly. **Conclusions**: Although anatomically close to the MNI, the EAM demonstrated inferior radiographic clarity and reproducibility. These findings suggest that the EAM may have limitations as a cranial reference landmark.

## 1. Introduction

The external auditory meatus (EAM) has long been employed as a practical surrogate landmark for the cranial center in radiographic and biomechanical studies of spinal alignment. This convention originates from classic cadaveric suspension studies, which defined the anatomical cranial center as the midpoint of the nasion–inion line (MNI), with the EAM serving as a close radiographic approximation of this midpoint [1,2]. In their cadaveric suspension experiment, Beier et al. reported in 1979 that the cranial center of gravity was located approximately 2.2–4.3 cm above the Frankfort plane and 0.2–1.3 cm anterior to the axis connecting the external auditory meatus [1]. Subsequently, Vital et al. reported in 1986 that the cranial center was situated near the midpoint of the nasion–inion line, posterior to the sella turcica and slightly superior and anterior to the external auditory meatus [2]. Based on these anatomical observations, the EAM has since been widely adopted as a practical cranial reference landmark in numerous studies investigating sagittal alignment, particularly in the evaluation of adult spinal deformity [3,4,5,6,7]. Most studies that define the external auditory meatus (EAM) as an approximation of the cranial center rely on the two aforementioned cadaveric suspension studies as the primary anatomical basis for this convention [1,2]. In addition, research evaluating global sagittal alignment and the relationship between cranial landmarks and the gravity line in a normal population reported that when the EAM was used as the head center, it was located very close to the gravity line, suggesting that the EAM may reasonably approximate the physiological head center [6]. These findings have further supported the widespread use of the EAM as a cranial reference point in sagittal balance analysis.

**Figure 1:**
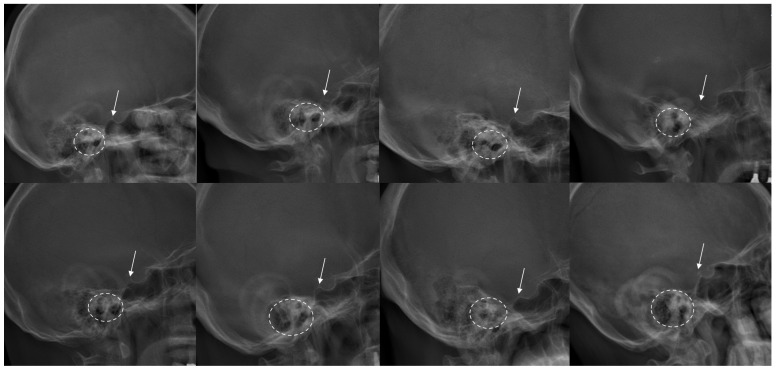
Various examples of patients’ head images in preoperative whole-spine X-rays. The posterior border of the sella turcica is indicated by a white arrow, while the external auditory meatus is indicated by a dotted circle. In most patients, the sella turcica appears clear and singular, while the external auditory meatus appears vague or duplex.

However, the radiographic clarity and consistency of the EAM can sometimes be limited in clinical imaging. These limitations arise largely from the presence of extensive mastoid air-cell pneumatization and the frequent occurrence of double-contour overlap in lateral radiographic projections (Figure 1). In addition, variations in head positioning, particularly subtle head rotation, may further obscure the margins of the EAM on whole-spine radiographs. Although the EAM approximates the head center and remains practically useful in many studies, insufficient clarity and consistency may limit its applicability in certain clinical situations. Despite its widespread clinical and research utilization as a cranial reference landmark, no studies to date have quantitatively evaluated the radiographic clarity of the EAM.

The aim of the present study was therefore to evaluate the clarity of the external auditory meatus (EAM) as a reference landmark for the cranial center, with particular emphasis on its radiographic visibility and interobserver agreement. In order to provide a comparative reference, the sella turcica was selected as a control landmark. The sella turcica is a saddle-shaped depression located on the superior surface of the sphenoid bone at the skull base [8,9]. It represents a singular, centrally positioned anatomical structure that is less frequently obscured by surrounding anatomical features and is generally less prone to double-contour overlap, even on radiographs that are not perfectly true lateral views. Because of these characteristics and its anatomical proximity to the EAM, the sella turcica was considered an appropriate control landmark for comparison. Accordingly, we compared the radiographic clarity of the EAM with that of the sella turcica in order to determine whether the EAM can serve as a sufficiently reproducible reference point for identifying the cranial center in patients with spinal deformity.

## 2. Materials and Methods

This retrospective study was conducted at a single tertiary referral institution and included patients who underwent surgical correction for sagittal spinal deformity. The study protocol was reviewed and approved by the Institutional Review Board, which also granted a waiver of informed consent because of the retrospective nature of the study (IRB No. 3-2025-0312). Patients who underwent surgery between January 2021 and December 2024 were screened for eligibility. The inclusion criteria for sagittal spinal deformity were defined according to commonly accepted radiographic parameters as follows: age greater than 18 years, sagittal vertical axis (SVA) greater than 5 cm, pelvic tilt greater than 25°, or thoracic kyphosis greater than 60°. Patients who did not have preoperative standing whole-spine radiographs available for analysis were excluded from the study.

All preoperative radiographic analyses were performed using EOS X-ray imaging, a three-dimensional imaging system that enables simultaneous acquisition of full-body images in the standing position while minimizing geometric distortion. During EOS image acquisition, the radiologic technician ensured that the patient’s head was not rotated and confirmed that the patient maintained a horizontal gaze before the scan was performed, following the standard imaging protocol used for spinal deformity evaluation. For each patient, two cranial reference landmarks were identified on the radiographs: the external auditory meatus (EAM) and the posterior border of the sella turcica. The nasion was defined as the most anterior point of the frontonasal suture, whereas the inion was defined as the most prominent posterior projection of the external occipital protuberance. The midpoint of the nasion–inion line (MNI) was then determined as the geometric midpoint between these two landmarks. Horizontal and vertical distances from each reference point to the MNI were measured in order to evaluate the spatial relationship between the cranial landmarks.

**Figure 2:**
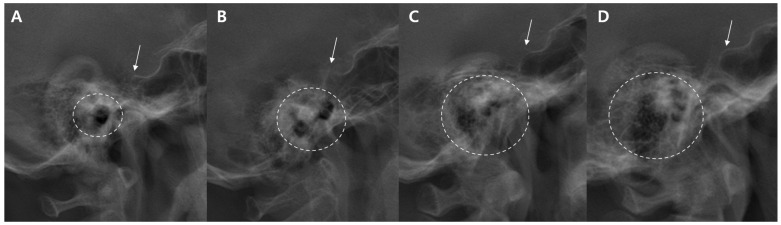
Examples illustrating the classification of the clarity of the reference point. The external auditory meatus (indicated by a dotted circle line) is categorized as (**A**) single point and clear, (**B**) duplex but distinguishable, or (**C**,**D**) unclear. In all four examples, the sella turcica (indicated by a white arrow) appears as a single and clearly identifiable point.

The radiographic clarity of each reference point was categorized into three groups: (1) single point and clear, when the landmark appeared as a well-defined single point; (2) duplex but distinguishable, when two potential points were visible but the landmark could still be reasonably identified; and (3) unclear, when the landmark could not be confidently identified because of radiographic overlap or poor visibility (Figure 2). All measurements were performed using a digital radiographic analysis software system (INFINITT PACS version 7.0; Infinite Healthcare, Seoul, Republic of Korea). Observers used standardized magnification and calibration settings to minimize measurement variability across assessments.

Measurements were independently performed by five board-certified neurosurgeons with experience in spinal deformity radiographic evaluation. Prior to performing the measurements, all observers were provided with a standardized protocol that described the definitions of the landmarks and the measurement procedures in detail. Each observer conducted the measurements independently and was blinded to the results obtained by the other observers. Each reference point was quantified using both horizontal and vertical coordinates relative to the MNI. Interobserver reproducibility was assessed using the intraclass correlation coefficient (ICC) calculated with a two-way random-effects model [ICC(2,1)]. To further evaluate measurement variability among observers, two additional parameters were calculated. The mean deviation among observers was defined as the average of the measurement differences between observers, representing the overall variability in landmark identification. The major deviation among observers was defined as the largest measurement difference observed among all observers, representing the maximum discrepancy in landmark localization.

Statistical analyses were performed using Python (version 3.12.2; Python Software Foundation). Continuous variables are presented as mean ± standard deviation, and categorical variables are presented as counts and percentages. Between-group comparisons were conducted using the Mann–Whitney U test for continuous variables and Fisher’s exact test or the chi-square test for categorical variables, as appropriate.

## 3. Results

**Figure 3:**
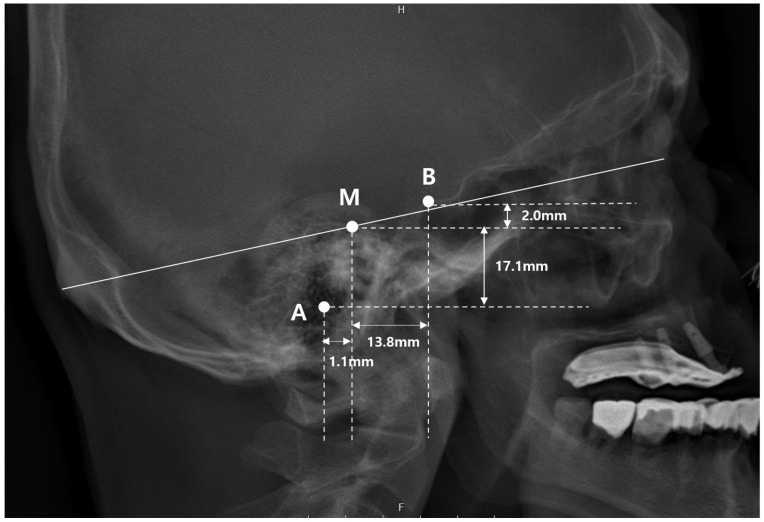
Illustration of the average spatial relationship among the MNI, EAM, and sella turcica. A: center of the external auditory meatus (EAM), B: posterior border of the sella turcica (SC), M: midpoint of the nasion–inion line (MNI). The EAM was located an average of 1.1 mm posterior and 17.1 mm inferior to the MNI. The SC was located an average of 13.8 mm anterior and 2.0 mm superior the MNI.

A total of 71 patients met the inclusion criteria and were included in the final analysis. The demographic characteristics and measurement parameters are summarized in Table 1. The external auditory meatus (EAM) was located horizontally closer to the midpoint of the nasion–inion line (MNI) than the posterior border of the sella turcica. On average, the EAM was positioned 1.1 mm posterior and 17.1 mm inferior to the MNI, whereas the posterior border of the sella turcica was located 13.8 mm anterior and 2.0 mm superior to the MNI (Figure 3). These measurements illustrate the relative spatial relationship between the EAM, the sella turcica, and the MNI within the cranial reference framework.

With respect to radiographic clarity, the sella turcica demonstrated superior visibility compared with the EAM. A total of 84.5% of sella turcica points were classified as single point and clear, whereas only 14.1% of EAM points met this criterion. Among EAM measurements, 47.9% were categorized as duplex but distinguishable, and 38.0% were classified as unclear. In contrast, 15.5% of sella turcica assessments were judged as unclear.

Interobserver reproducibility analysis demonstrated that measurements of the sella turcica showed higher agreement among observers compared with those of the EAM. The intraclass correlation coefficient (ICC) for the sella turcica was 0.97 (95% CI, 0.96–0.98) in the horizontal dimension and 0.95 (95% CI, 0.93–0.97) in the vertical dimension, indicating excellent interobserver agreement. In comparison, the EAM demonstrated lower reproducibility, particularly in the horizontal dimension, with an ICC of 0.84 (95% CI, 0.80–0.88) horizontally and 0.89 (95% CI, 0.85–0.93) vertically.

Measurement variability among observers further demonstrated greater dispersion for the EAM compared with the sella turcica. The mean deviation among observers for EAM measurements was 2.6 ± 1.4 mm horizontally and 1.8 ± 1.9 mm vertically, whereas the corresponding values for the sella turcica were 0.9 ± 0.6 mm horizontally and 1.6 ± 1.2 mm vertically. The major deviation among observers showed a similar pattern. For the EAM, the major deviation was 6.3 ± 3.5 mm horizontally and 4.3 ± 4.7 mm vertically, whereas for the sella turcica it was 2.2 ± 1.5 mm horizontally and 3.9 ± 2.8 mm vertically.

**Figure 4:**
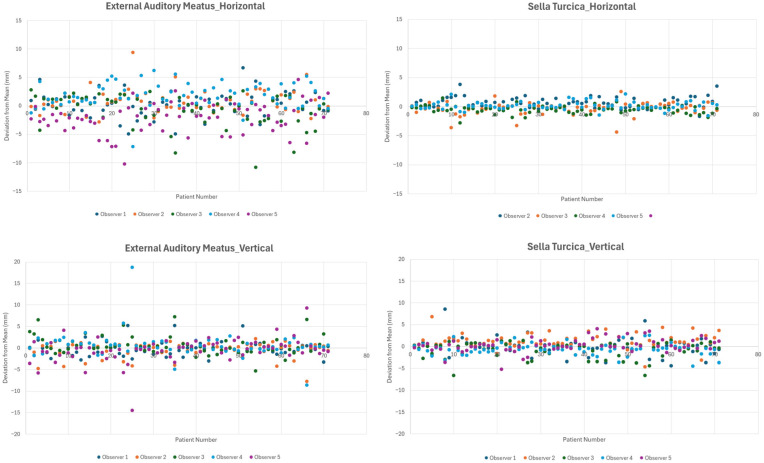
Scatter plots showing the measurements of the external auditory meatus and the sella turcica for each patient and each observer. Each data point represents the horizontal or vertical deviation from the mean value of all observers. The external auditory meatus demonstrated greater variability than the sella turcica, particularly in the horizontal dimension.

Statistical comparison revealed that the horizontal deviation among observers was significantly greater for the EAM than for the sella turcica (*p* < 0.001) in both the mean deviation and major deviation analyses. In contrast, vertical deviations did not differ significantly between the two reference points (*p* = 0.985 and *p* = 0.934, respectively). As illustrated in the scatter plot of deviations from the mean observer values, the EAM demonstrated greater dispersion among observers, particularly along the horizontal axis, whereas the sella turcica measurements showed relatively tighter clustering around the mean values (Figure 4).

## 4. Discussion

In this study, we sought to assess the radiographic clarity of the external auditory meatus (EAM) by assessing its interobserver reproducibility. Although the EAM was horizontally located closer to the midpoint of the nasion–inion line (MNI) than the sella turcica (1.1 mm vs. 13.8 mm), our analysis demonstrated that the EAM exhibited somewhat greater interobserver variability and a higher rate of being classified as duplex or unclear (85.9% vs. 15.5%). This limitation was especially pronounced along the horizontal axis, where the major deviation among observers was 6.3 ± 3.5 mm on average, exceeding the level of ambiguity generally considered acceptable for radiographic measurements. These findings suggest that the clarity of the EAM may pose significant constraints, particularly when evaluating sagittal alignment in lateral radiographs for patients with spinal deformity.

It is well recognized that the visibility of various anatomical reference points poses inherent limitations for radiographic measurements. This issue becomes even more pronounced in patients with spinal deformity, where combined coronal and sagittal malalignment make it challenging to obtain true lateral or anteroposterior radiographs. For example, the center of the femoral head is commonly used for assessing pelvic parameters; however, the two femoral heads often do not align perfectly in a true lateral projection and appear as two overlapping contours. In such cases, because each femoral head is still a clearly identifiable structure, the midpoint between the centers of the two heads is used as an approximation [10], and this approach is generally considered acceptable.

In contrast, the external auditory meatus (EAM) presents considerably more complex visibility challenges. It not only appears duplicated in a large proportion of cases due to slight rotational differences or imperfect lateral projection, but its true margins are also frequently obscured by the overlying mastoid air cells. As a result, even experienced observers may struggle to consistently identify the exact anatomic location of the EAM on standing whole-spine radiographs. These limitations were reflected in both our interobserver reliability analysis and scatter plots, especially along the horizontal axis. The magnitude of this variation exceeded what is generally regarded as an acceptable tolerance for radiographic measurement error, implying a limitation of the EAM as a reproducible cranial reference point in patients with spinal deformity.

In our results, the dispersion of the EAM in the vertical axis was not substantially greater than that of the sella turcica; however, a noticeably larger dispersion was observed in the horizontal axis. During whole-spine radiographic acquisition, technicians typically follow standardized protocols, such as instructing the patient to maintain a horizontal gaze. Nevertheless, particularly in patients with spinal deformity, some degree of unavoidable head rotation may occur. Under such circumstances, the EAM is likely to be more susceptible to positional changes, which may explain the increased horizontal dispersion observed in our measurements. When analyzing sagittal alignment, if a reference point demonstrates large horizontal dispersion, the reliability of angle or distance measurements derived from that reference point inevitably decreases.

In spinal deformity research, the cranial center is commonly considered to correspond to the midpoint of the nasion–inion line (MNI), and the external auditory meatus (EAM) is widely used as a practical surrogate for this point because of its close anatomical proximity. In many recent spinal deformity studies, parameters related to the cranial center have been analyzed using the MNI as the representative reference point, and meaningful radiographic and clinical findings have been reported based on this approach [4,5,7]. The role of the EAM as a cranial center in patients with deformity has also been established in numerous studies. In one study analyzing sagittal alignment in a normal population, the gravity line was shown to pass exactly through the EAM, suggesting that the EAM should correspond closely to the physiological center of the head [6]. One study used the anterior border of the EAM as the cranial center of gravity, demonstrating that the horizontal distance between the EAM and the posterior superior iliac spine (PSIS) showed a strong correlation with health-related quality of life (HRQOL) in patients [11]. Accordingly, many studies have used the EAM as a surrogate indicator of the cranial center of gravity, and radiographic as well as clinical findings derived from this reference have been shown to be meaningful and well supported [12,13,14].

However, we should recognize that the EAM may not be an exact representation of the true cranial center. Makino et al. demonstrated that the MNI and EAC locations had horizontal differences, and implicated that the sagittal vertical axis should be interpreted with consideration of the differences between the MNI and the EAC [15]. A cadaveric study conducted in Germany in 1979 reported that the center of gravity of the head is located within the midsagittal plane, 0.8 cm in front of the EAM and 3.1 cm above the Frankfort plane [1]. A 1986 cadaveric study from France described the center of gravity as most frequently located at the midpoint of the nasion–inion line, which lies behind the sella turcica, above and slightly in front of the external auditory meatus [2]. In fact, it is unlikely that a single reference point that perfectly corresponds to the true cranial center exists, as subtle anatomical variations are inevitably present between individuals. The ideal center of mass should project near the center of the support area formed between the two feet during a stable standing posture, where only minimal and constant balancing efforts are required [16]. Based on existing cadaveric literature, the midpoint of the nasion–inion line (MNI) is likely the landmark that most closely approximates the true cranial center. Accordingly, many spinal deformity studies have adopted the MNI itself as the cranial reference point and have reported meaningful results using this approach [4,5,7]. Although the present study did not evaluate the clarity and reproducibility of the nasion and inion, these landmarks may also appear obscure on radiographs depending on head rotation. Furthermore, determining the midpoint between two landmarks would inevitably introduce additional interobserver variability. Ideally, a single-point reference landmark that closely approximates the cranial center would be preferable; however, the present study raises the question of whether the EAM can fulfill this role in terms of clarity and reproducibility.

Thus, the EAM serves only as an approximation of the cranial center and has been widely adopted primarily because it represents a readily identifiable landmark on lateral radiographs. In our spatial analysis, the sella turcica was located on average roughly 1.5 cm anterior to the EAM, suggesting that the true cranial center likely lies somewhere between these two structures. Furthermore, given that the sella turcica is vertically much closer to the MNI, it may be positioned more closely to the true cranial center in the vertical dimension. This suggests that while the EAM may be acceptable as a cranial reference point when evaluating horizontal distances, such as in the case of sagittal vertical axis-type parameters, it may be less appropriate as a surrogate for the cranial center when measuring angles relative to other reference points. Parameters involving the sella turcica have recently been proposed for angular measurements. Le Huec et al. described the spino-cranial angle (SCA), defined as the angle formed between the tangent to the superior endplate of C7 and the line connecting the center of the sella turcica to the midpoint of the C7 endplate [17,18,19]. In an asymptomatic population, economic sagittal balance was characterized by a relatively constant SCA of 83 ± 9°. More recent studies investigating this parameter have further demonstrated its clinical utility; for example, an SCA of less than 79.1° has been reported as a useful indicator for detecting degenerative cervical spondylolisthesis [20,21].

The sella turcica is a saddle-shaped depression located on the superior surface of the sphenoid bone at the base of the skull [8,9]. It houses the pituitary gland, making it a central landmark in neuroanatomy and neuroradiology. The structure is bordered anteriorly by the tuberculum sellae and posteriorly by the dorsum sellae, with the hypophyseal fossa forming the central concavity. Because of its deep midline position and its consistent visibility on lateral skull and whole-spine radiographs, we chose the sella turcica as a control reference point while validating the EAM. Its anatomical stability and clear osseous margins should have contributed to excellent interobserver reproducibility compared with other variable external cranial landmarks. The sella turcica is also currently used in spinal alignment research through the parameter known as the spino-cranial angle (SCA), which is defined as the angle formed between the tangent to the superior endplate of C7 and the line connecting the center of the sella turcica to the midpoint of the C7 endplate [22]. Nevertheless, whether various sagittal parameters referenced to the sella turcica should prove clinically meaningful in deformity patients requires additional future studies.

The primary aim of this study was to evaluate the radiographic clarity of the EAM and its reproducibility among observers. Although our findings indicate that the EAM may lack some reproducibility, whether it should be replaced by an alternative cranial reference point is more complex and warrants further investigation. While several anatomical studies have suggested that the EAM is the closest and most practical surrogate for the true cranial center, these theoretical advantages may not fully translate into radiographic practice. In real-world clinical imaging, the EAM may suffer from duplication, obscuration by surrounding structures, and inconsistent visibility, all of which can introduce interobserver ambiguity. Therefore, the radiographic ambiguity associated with the EAM may limit its utility, highlighting the need for additional studies that explore alternative landmarks or imaging techniques capable of providing both anatomical relevance and consistent measurability.

This study has several limitations. First, this is a retrospective analysis, and the possibility of selection and observational bias cannot be completely excluded. Second, the cohort consisted solely of patients with sagittal spinal deformity from a single tertiary center, which may restrict generalizability to other populations or imaging settings. Because the analysis was conducted only in patients with spinal deformity and did not include a control group of non-deformity individuals representing a relatively normal population, there are limitations in validating the findings. Third, although five observers participated, differences in experience or interpretation of ambiguous contours may still have contributed to measurement variability. Additional statistical analyses that could further strengthen the results, such as intra-observer reliability and power analysis, were not performed in the present study. In addition, this study did not include the evaluation of other cranial landmarks or sagittal parameters, such as the sagittal vertical axis referenced to the EAM or the sella turcica. Finally, the clinical application or impact of the observed measurement variability on sagittal alignment parameters was not directly evaluated. The present work should be interpreted as a pilot study for assessing the radiographic clarity and reproducibility of the EAM. Future studies performed in large numbers and multi-modalities are needed to further clarify and validate the optimal cranial reference point for sagittal alignment assessment.

## Figures and Tables

**Table 1:** Summary of measurements from 71 deformity patients.

Demographic Variable	Total Patients (*n* = 71)		
	External Auditory Meatus	Sella Turcica	*p*-Value
**Age**	66.0 ± 8.7		
**Female**	50 (70.4)		
**Distance from MNI (mm)**			
Horizontal	−1.1 ± 5.0	13.8 ± 3.6	
Vertical	−17.1 ± 5.3	2.0 ± 6.0	
**Clarity of identification**			<0.001
Single point and clear	10 (14.1)	60 (84.5)	
Clear but duplex	34 (47.9)	0 (0.0)	
Unclear	27 (38.0)	11 (15.5)	
Interobserver Reliability *			
Horizontal	0.84 (0.80–0.88)	0.97 (0.96–0.98)	
Vertical	0.89 (0.85–0.93)	0.95 (0.93–0.97)	
**Deviations** **among observers**			
Mean deviation (mm)			
Horizontal	2.6 ± 1.4	0.9 ± 0.6	<0.001
Vertical	1.8 ± 1.9	1.6 ± 1.2	0.985
Major deviation (mm)			
Horizontal	6.3 ± 3.5	2.2 ± 1.5	<0.001
Vertical	4.3 ± 4.7	3.9 ± 2.8	0.934

Continuous variables are depicted as mean ± SD and categorical values as number (%). MNI = midpoint of the Nasion–Inion line; * Calculated using the intraclass correlation coefficient, with a two-way random-effects model [ICC(2,1)].

## Data Availability

The data supporting the findings of this study are not publicly available due to privacy and ethical restrictions but are available from the corresponding author upon reasonable request.

## Abbreviations

The following abbreviations are used in this manuscript:

EAM	External Auditory Meatus
MNI	Midpoint of Nasion–Inion line
SVA	Sagittal Vertical Axis
ICC	Intraclass Correlation Coefficient
